# Magnetic Solid-Phase Extraction Based on Silica and Graphene Materials for Sensitive Analysis of Emerging Contaminants in Wastewater with the Aid of UHPLC-Orbitrap-MS

**DOI:** 10.3390/molecules28052277

**Published:** 2023-02-28

**Authors:** Maria Kalaboka, Vasilios Sakkas

**Affiliations:** Chemistry Department, University of Ioannina, 45110 Ioannina, Greece

**Keywords:** MSPE, magnetic materials, graphene oxide, LC-Orbitrap-MS, pharmaceutical compounds, artificial sweeteners, sample preparation

## Abstract

With the advancement of technology and nanotechnology, new extraction sorbents have been created and effectively used for the magnetic solid-phase extraction of target analytes. Some of the investigated sorbents have better chemical and physical properties, exhibiting high extraction efficiency and strong repeatability, combined with low detection and quantification limits. In this study graphene oxide (GO) magnetic composites were prepared and used as magnetic solid-phase extraction (MSPE) adsorbents along with synthesized silica based magnetic nanoparticles (MNPs) functionalized with the C18 group for the preconcentration of emerging contaminants (ECs) in wastewater samples generated from hospital and urban facilities. The sample preparation with magnetic materials was followed by UHPLC-Orbitrap MS analysis for the accurate identification and determination of trace amounts of pharmaceutical active compounds and artificial sweeteners in effluent wastewater. Optimal conditions were used for the extraction of ECs from the aqueous samples, prior to UHPLC-Orbitrap MS determination. The proposed methods achieved low quantitation limits between 1.1–33.6 ng L^−1^ and 1.8–98.7 ng L^−1^ and satisfactory recoveries in the range of 58.4%–102.6%. An intra-day precision of less than 23.1% was achieved, while inter-day RSD% values in the range of 5.6–24.8% were observed. These figures of merit suggest that our proposed methodology is suitable for the determination of target ECs in aquatic systems.

## 1. Introduction

Emerging contaminants (ECs) encompass a vast group of chemical compounds appearing in industrial and agricultural activities as well as in daily lifestyle habits. They are a new class of unregulated pollutants and have attracted scientific interest. ECs consist of a heterogenous group of chemicals that include pharmaceutical active compounds (PhACs) and personal care products (PCPs), pesticides, artificial sweeteners (ASs), plasticizers, flame retardants, perfluorinated organic compounds (PFOs), etc. [1].

The main pathway of the continuous discharge of emerging contaminants such as ASs, PhACs, and PPCPs into the natural aquatic environment is wastewater treatment plants (WWTPs), which they reach either as excretion products from urine and feces or as a result of direct discharge, manufacturing processes, etc. Conventional WWTPS seem inadequate to completely prevent these types of pollutants of being transported, through final effluents, into the aquatic environment. ECs are frequently detected in both influent and effluent water originating either from hospital treatment units or urban plants at concentration levels of ng/L and μg/L [2,3,4]. The occurrence of ECs in such low concentration levels, along with the complexity of the matrix of a wastewater sample makes their analysis and determination quite challenging. To address this issue, innovative technologies, chromatographic materials and analytical approaches focusing on selectivity, sensitivity, and simplicity of an analytical method are required. Liquid chromatography–mass spectrometry (LC–MS), and particularly LC–tandem mass spectrometry (MS/MS), provides very low determination limits and has been the main technique for quantifying pharmaceutical residues and artificial sweeteners in wastewater. Upgraded LC-MS technologies have been put forward in recent years to try and meet the requirements for the accurate identification of multiclass compounds with diverse groups, especially in such complex environmental samples [3,4].

The analysis of contaminants of emerging concern in wastewater environmental samples mainly involves the preconcentration and cleanup of the influents or/and effluents before their instrumental analysis. Solid phase extraction (SPE) has replaced conventional extraction techniques, such as traditional liquid–liquid extraction (LLE), and is the main technique of choice for the multiresidue analysis of pharmaceutical active compounds and personal care products. Recent trends in sample preparation focus on the miniaturization of the process, the development of new sorbent materials, and the enhancement of environmentally friendly approaches. Among these techniques magnetic solid phase extraction (MSPE) has gathered considerable attention in the last decade with an increasing number of publications describing the synthesis and application of different magnetic nanoparticles (MNPs) as sorbent materials. Included among the MSPE applications are the determination of drugs in biological and environmental matrices [5,6], food analysis [7,8], as well as the trend of the last decade, water treatment. The implementation of nanomaterials in water treatment and especially in the removal of ECs has gained great interest in environmental monitoring studies [9,10]. In this method, magnetic sorbents are added and dispersed in the sample, the analytes of interest are adsorbed onto their surface, and the material is separated easily with the application of a magnetic field. Subsequently, with the addition of a suitable organic solvent, the analytes of interest are eluted and preconcentrated, while using magnetic separation the liquid phase is collected for further analysis. The main benefits of MSPE are the direct addition of the sorbent in the sample; the high dispersion of the applied materials overcoming packing issues, such as blocked cartridges; the rapid and simple separation of sorbent materials without further filtration or centrifugation steps; and the decreased consumption of organic solvents compared to traditional SPE and LLE techniques [11]. The aforementioned significant advantages have led to the development of MSPE techniques with the fabrication of a vast group of materials that can be employed as solid sorbents. These materials usually consist of magnetic nanoparticles based on a magnetic core of metal oxides (e.g., Fe_3_O_4_) coated with polymers, silica, metal-organic frameworks (MOFs), multiwalled carbon nanotubes (MWCNTs), and graphene (G) or graphene oxide (GO). One of the most frequently used magnetic materials is silica based (Fe_3_O_4_@SiO_2_) being functionalized with different moieties, such as the C18 groups (Fe_3_O_4_@SiO_2_-C18) [12,13]. Fe_3_O_4_@SiO_2_-C18 magnetic nanoparticles have been employed as magnetic sorbents for a wide range of MSPE applications, including the preconcentration of drugs in biological samples [14], the determination of residual traces of pesticides in environmental waters [15], food sample preparation for the analysis of antibiotics [16], etc. Other sorbents that have captured attention in material science and have been widely employed in MSPE in recent years are carbon-based materials and especially graphene and graphene oxide or reduced graphene oxide (rGO) composites. GO is the oxidized form of graphene which can be easily fabricated from natural graphite according to Hummers and Offeman’s method, while rGO is a GO derivative, partly reduced. Taking advantage of the benefits of GO as a sorbent material and the convenience of magnetic separation when combined with MNPs, magnetic graphene-based nanocomposites were fabricated for MSPE sorbents for the sample preparation of biological matrices (urine, plasma), in order to determine drugs and molecules of biological interest, such as pseudoephedrine, psychoactive drugs, flavonoids, methamphetamine, and monohydroxy polycyclic aromatic hydrocarbons. According to authors’ knowledge it is the first time that MSPE extraction has been combined with high-accuracy mass spectrometric tools, and especially Orbitrap MS, for the determination of 33 multiclass emerging contaminants belonging to diverse group categories (antibiotics, non-steroid anti-inflammatory drugs, antidepressant and antipsychotic drugs, disinfectants, lipid regulators, and artificial sweeteners) in environmental samples.

## 2. Results

### 2.1. UHPLC-Orbitrap MS

The effective separation of 33 target analytes was accomplished after the evaluation of the optimal chromatographic conditions (chromatographic column, mobile phase, etc.). A typical chromatogram of 33 analytes of interest is illustrated in Figure S1. The instrumental detection limits, mass accuracy and fragment ions, and their corresponding collision energy for all the emerging contaminants of the study in positive and negative ionization modes are included in Tables S2 and S3 in the supplementary material.

### 2.2. Characterization of Synthesized Materials

The characterization results concerning Fe_3_O_4_, Fe_3_O_4_@SiO_2_, and Fe_3_O_4_@SiO_2_@C18 nanomaterials are presented in a previously published work of the authors [17].

#### 2.2.1. Graphene-Based (GO) Composites

The synthesized composites of reduced graphene oxide modified by magnetic iron oxide (mrGO), the intermediate materials of graphene oxide (GO), reduced graphene oxide (rGO), and the magnetic composite of graphene oxide were characterized with the X-ray diffraction technique (XRD), scanning electron microscopy (SEM), and Fourier transform-infrared spectroscopy (FT-IR).

##### X-ray Diffraction Technique (XRD)

**Figure 1:**
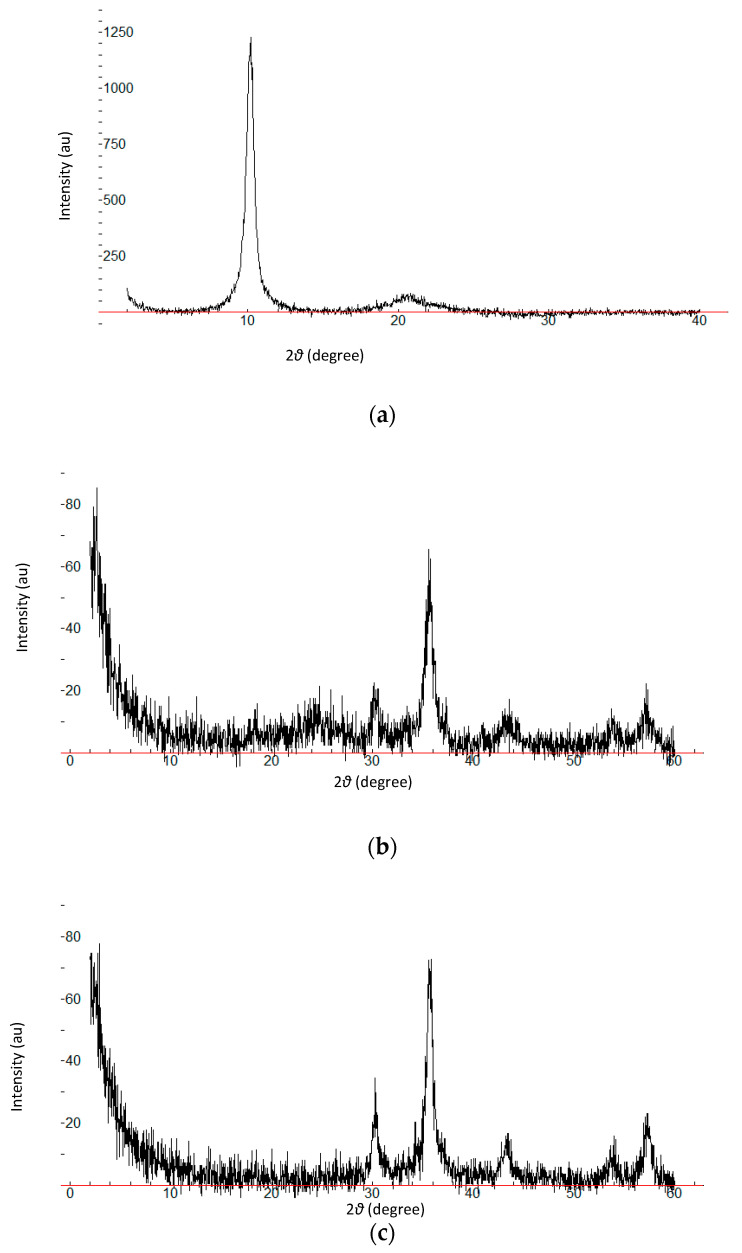
XRD patterns of (**a**) GO, (**b**) rGO, and (**c**) mrGO. X axis:2θ (degree), Y axis: Intensity (au).

XRD measurements were employed to investigate the crystalline phase and structure of the synthesized materials. Figure 1a shows the XRD patterns of GO, rGO, mGO, and mrGO. The peak observed at 2θ = 10.19° of GO was attributed to the introduction of oxygen-containing functional groups into the graphite sheets in the formation of GO [18,19]. These functional groups facilitated the hydration and exfoliation of GO in water. This peak, along with the disappearance of the intensive diffraction peak at 25.5° of graphite (Figure 1b), is indicative of the total oxidation of graphite to graphene oxide. For the rGO pattern, a weak and broad reflection peak was observed at 24.44°, which can be ascribed to the relative short-range order structures in disordered stacked rGO [20,21], which implies the successful reduction of GO. Five diffraction lines were observed in the representative XRD patterns of mGO and mrGO at 2θ 30.14°, 35.84°, 43.73°, 53.63°, 57.26°, and 30.11°, 35.75°, 43.55°, 53.96°, 57.35°, respectively (Figure 1c). These characteristic diffraction peaks match the cubic spine crystal structure of iron oxide, suggesting the existence of Fe_3_O_4_ [22,23].

##### FT-IR Spectroscopy of Magnetic Graphene Based Nanocomposites

**Figure 2:**
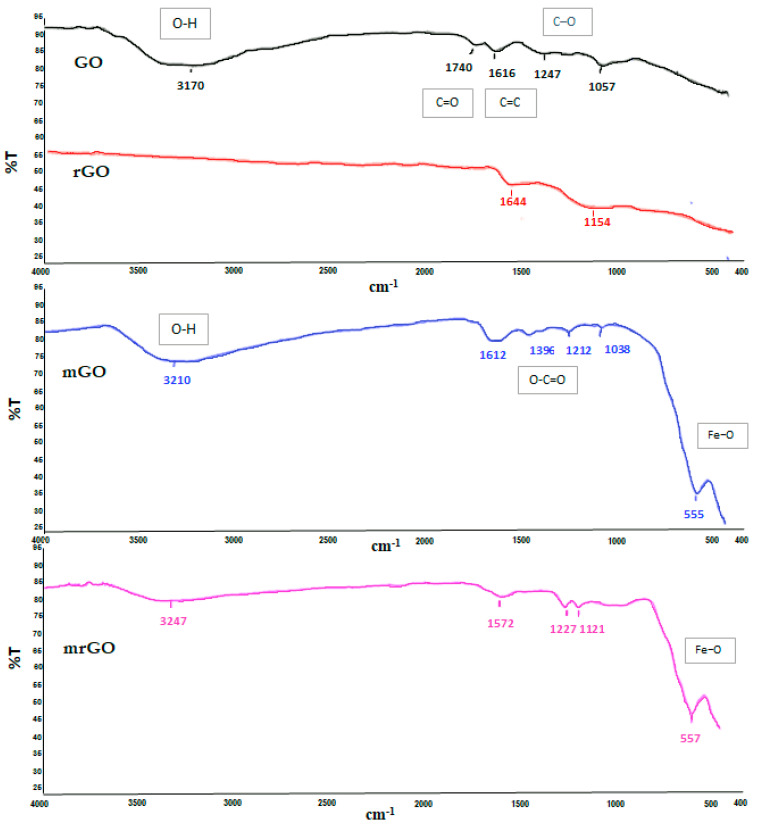
FT-IR spectra of GO, rGO, mGO, and mrGO.

Fourier transform infrared spectroscopy (FT-IR) spectra provide information about the functional groups of the synthesized materials. The FT-IR spectra of the prepared GO based materials are shown in Figure 2. The GO and mGO spectra are characterized by a broad peak at 3170 cm^−1^ and 3210 cm^−1^, respectively, which is assigned to the O–H stretching vibrations of the hydroxyl group. The peak at 1740 cm^−1^ corresponds to the carbonyl or carboxyl groups (C=O), and that at 1616 cm^−1^ refers to the aromatic C=C bonds. Moreover, the C–O stretching vibrations of the epoxy group and alkoxy were observed at 1247 cm^−1^ and 1057 cm^−1^, respectively [24]. These characteristic bands in the GO spectrum were attributed to the oxidation process which has introduced strong oxygen-containing functional groups in the initial graphite. On the other hand, all the absorption bands related to the oxidized groups were significantly diminished or even disappeared in the FT-IR spectrum of rGO, indicating the successful reduction of graphene oxide. In the FT-IR spectrum of mGO, adsorption bands that characterized the GO spectrum were also observed, but the positions of the bonds were slightly shifted and the sharpness of the peaks has changed, indicating the change in the coordination environment of various functional groups in mGO [25]. An additional peak observed at 1396 cm^−1^ can be assigned to O–C=O carboxyl bonds (COO-symmetric vibration). Finally, in the low frequency region, a new peak appeared at 555 cm^−1^ which corresponds to the stretching vibration of the Fe−O, implying that Fe_3_O_4_ is attached with the O–C=O—group on the edge of the GO [26]. As far as concerns the FT-IR spectrum of mrGO (Figure 2), in accordance with rGO, the decrease of the peaks attributed to oxygen-containing functional groups confirms the reduction. The peaks at 1227 cm^−1^ and 1121 cm^−1^ correspond to the epoxy and alkoxy groups, while the peak at 1574 cm^−1^ is attributed to the aromatic C=C bonds. Finally, as for mGO, the existence of Fe_3_O_4_ is confirmed by the strong band at 557 cm^−1^, characteristic of the Fe–O bond.

##### Scanning Electron Microscopy (SEM) of GO and mrGO

**Figure 3:**
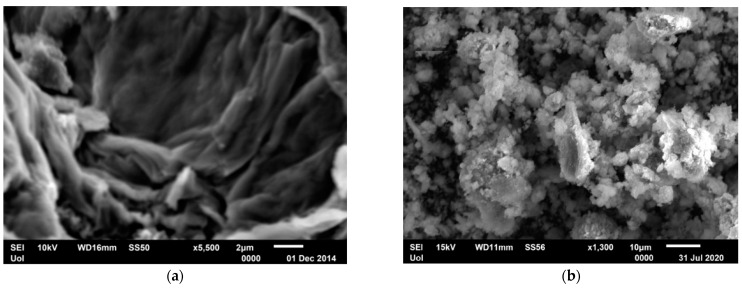
SEM images of (**a**) graphene oxide (GO) and (**b**) mrGO.

Figure 3 portrays the SEM images of graphene oxide (GO) at two magnitudes. The morphology of GO is observed as thin randomly orientated crumpled sheets, reflecting its layered structure. The characteristic wrinkled sheets of GO maintain a large surface area providing many adsorption sites. No amorphous or other kinds of crystallized phase particles are observed. On the other hand, in the SEM image of mrGO as depicted in Figure 3, some particles are distinguishable on the graphene layers and are attributed to the attached Fe_3_O_4_. Their shape is irregular spherical and the are arranged heterogeneously in bunches on the graphene layers due to the occurrence of some agglomeration. In any case, the microspheres are well integrated with the GO sheets, showing the successful synthesis of mrGO.

### 2.3. Optimization of Magnetic Solid-Phase Extraction (MSPE)

**Figure 4:**
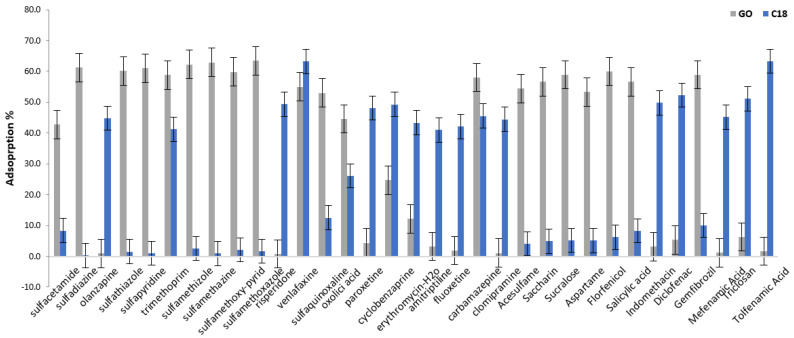
Sorption of target analytes in Fe_3_O_4_@GO (GO) and Fe_3_O_4_@SiO_2_@C18 (C18).

Some preliminary absorption experiments were conducted to investigate the sorption capacity of the synthesized materials on the target emerging contaminants (ECs). All the adsorption experiments were performed in a series of 15-mL tubes containing 10 mg of each magnetic sorbent (Fe_3_O_4_@GO and C18@SiO_2_@Fe_3_O_4_). Ten (10) mL of aqueous pharmaceutical standard solution with an initial concentration of 50 μg/L at a neutral pH was used. All the mixtures were placed in a shaker platform and were mixed for one hour at room temperature. After one hour the adsorbents were isolated from the solution using a strong magnet and the analytes were eluted with2 mL of methanol, applying 15 min of stirring. Finally, an aliquot of the eluent was injected in a UHPLC-Orbitrap-MS system. Figure 4 shows the sorption performances of the magnetic nanoparticles (MNPs), Fe_3_O_4_@GO and Fe_3_O_4_@SiO_2_@C18, towards selected analytes. All the experiments were conducted in triplicate. The average values were used.

Fe_3_O_4_@SiO_2_@C18 favors the sorption of non-polar compounds due to the function of the hydrophobic octadecyl group, while Fe_3_O_4_@GO provided mostly disappointing results. On the contrary, Fe_3_O_4_@GO favors the sorption of polar compounds. Hydrophilic functional groups, such as hydroxyl and carbonyl, increase the affinity of graphene with polar compounds. The extraction performance of these sorbents in relation to the polar analytes was in line with the relative polarity of the sorbents (the “like dissolves like” principle). Polarity data of the target analytes is presented in the supplementary material (Table S3). Only two compounds, trimethoprim and carbamazepine, had adequate sorption efficiency in both materials. For this reason, two different protocols, mentioned in the article as MSPE-Fe_3_O_4_@GO and MSPE Fe_3_O_4_@C18, were established for the determination of emerging contaminants in wastewater using two varied materials as sorbents in the MSPE procedure. Table S1 presents the sorbents and the corresponding analytes for each MSPE technique.

#### 2.3.1. Optimization of MSPE- Fe_3_O_4_@SiO_2_@C18

A one-variable-at-a-time optimization approach was employed for the optimization of MSPE conditions. All the optimization experiments were conducted in aqueous solutions fortified with 19 pharmaceuticals of 200 ng/L. The optimum conditions established were subsequently applied and tested in waste waters. Parameters such as the sample pH, sorbent amount, elution solvent, extraction and elution times, and sample volume were investigated to achieve the optimal extraction efficiencies for the 19 selected pharmaceuticals. The optimal conditions are displayed in Table 1.

##### Effect of the pH

**Figure 5:**
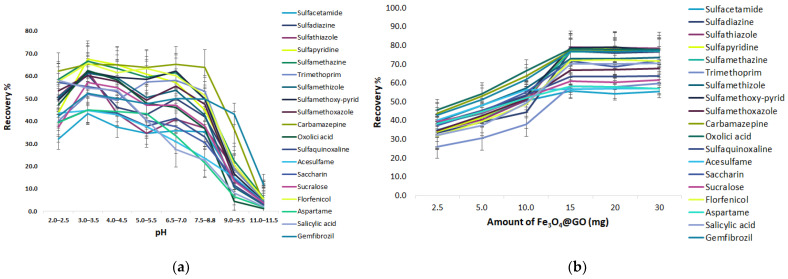

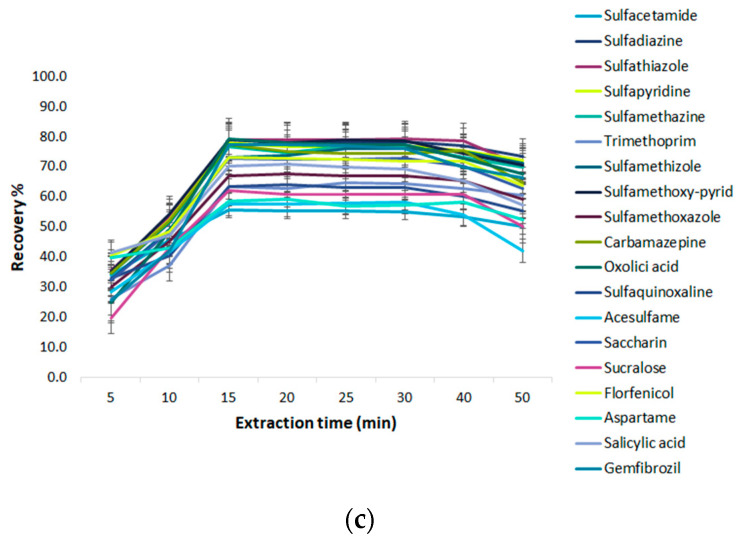
Effect of the extraction parameters of MSPE@GO (**a**) pH, (**b**) amount of GO@Fe_3_O_4_, (**c**) extraction time.

As shown in Figure 5a, the ECs gradually rise when increasing the pH from 2.0 to 3.0 and then decrease with the further increase of the pH from 4.0 to 6.0. At pH 6.5–7.0 a minor increase is presented, while the adsorption recovery of sulfonamides (SAs) decreased considerably when the pH peaked at a range from 8.5 to 11.5. The optimum pH for SA adsorption is 3.0–3.5; at pH values close to 3.0–3.5, the neutral and positively charged form of SAs co-exist according to their dissociation constant. In addition, the speciation of the GO functional group changes as well with pH variations. Typically, the alkyl carboxyl (COOH) functional group has a pKa of 4.5. Thus, at a pH of 3.0–3.5, almost all the carboxyl groups are protonated with natural charge (-COOH) [27].

##### Amount of GO@Fe_3_O_4_

To acquire the maximum extraction efficiency of the target analytes, different amounts of the magnetic graphene (2.5, 5, 10, 15, 20, 30 mg) were assessed. As shown in Figure 5b, the recoveries of the analytes increased with increasing sorbent doses from 2.5 to 15 mg, due to enhancement in the surface area and active sites, and then stabilized with no further increases. Substantial amounts of sorbent are useful for extraction but are inconvenient for the removal of the desorption solvent. Thus, 15 mg was employed in the next experiments.

##### Effect of Extraction Time on Fe_3_O_4_ @GO-MSPE

To enhance the extraction efficiency, the selection of extraction time is important after the sorbents are dispersed into the solution. A good dispersion of adsorbent is beneficial to the improvement of sorption efficiency in the least time, due to the large contact surface area between the adsorbent and analytes in the water sample. In this study, magnetic stirring was firstly used to assist the extraction of target analytes. The effect of stirring time on the extraction efficiency was investigated in the range of 1–50 min at an ambient temperature. The results in Figure 5c show that the extraction efficiency increased by increasing the extraction time from 1 to 10 min, and then the upward trend became gradually slower in the following five minutes. The extraction reached equilibrium and the recoveries of analytes nearly reached a maximum value when the extraction time was 15 min. Therefore, 15 min of extraction time was selected for the subsequent experiments.

##### Type of Desorption Solvent

Several elution solvents of different polarities were considered (methanol, acetone, and acetonitrile) to break down the π-π and hydrophobic interactions.

**Figure 6:**
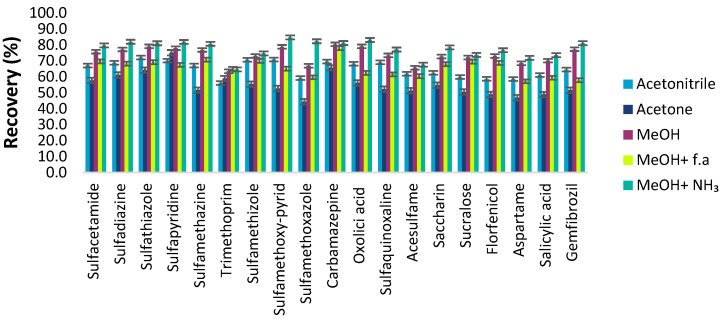
Effect of the desorption solvent on the recoveries of ECs.

The results in Figure 6 show that the efficiency of MSPE reached a maximum when methanol was used as the elution solvent under the same extraction and desorption conditions. To increase the desorption efficiency, taking into consideration the pH sorbent dependence, formic acid or ammonia was added at various percentages (1–5% *v*/*v*) to increase the acidity or alkalinity. The addition of formic acid (f.a) was not more efficient than pure methanol in most of the compounds, while on the other hand, the addition of ammonia significantly increased the recoveries of the extraction. This is reasonable and in accordance with the results from the pH optimization of sorption process, as well as with the physicochemical properties (PKa, logD) of the compounds, since it was found that an alkaline environment does not favor the sorption, resulting in an increase in the desorption efficiency. The effect of ammonia percentage in methanol was also studied in the range of 0–5% (*v*/*v*). The result indicated that the desorption efficiency of methanol containing 1% ammonia was superior to pure methanol. When increasing the percentage of ammonia more than 1%, the extraction recoveries of SAs decreased slightly. This is probably because SAs will be less positively charged in an alkaline medium, resulting in weakening the affinity with the sorbent [28]. Thus, methanol containing 1% ammonia (*v*/*v*) was selected as the optimum elution solvent.

##### Elution Volume and Desorption Time

The volume of elution solvent and the elution time are also principal factors to obtain reliable and reproducible analytical results. The desorption efficiency of target analytes increased with the increase of the desorption solvent volume in the range of 0.5–5.0 mL, and no obvious changes were observed when the volume was further increased from 4.0 mL. It was observed that all the analytes could be nearly completely desorbed from the sorbent by sonication for 1 min with 2.0 mL desorption solvent twice. The desorption time from 60 s to 240 s under sonication was studied. The result indicated that the sonication time had no clear effect on the eluting efficiency after 120 s. Thus, the desorption time selected was 120 min (2 × 60 s) and the solvent volume was 4 mL (2 × 2 mL).

##### Effect of Sample Volume

**Figure 7:**
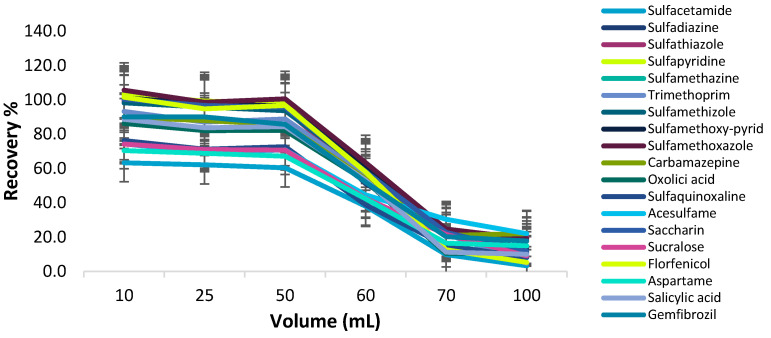
Effect of sample volume on Fe_3_O_4_ @GO-MSP.

To obtain the maximal extraction efficiency and lower MQLs the effect of the sample volume on the extraction efficiency was examined under the optimal conditions (10 mg of adsorbent, pH 3.0, 15 min of extraction time, 2 mL of methanol containing 1% NH_3_, and 60 s sonication twice). The different sample volumes (10–100 mL) investigated contained 1 μg of the target analytes. As shown in Figure 7, the target analytes provided satisfactory recoveries in a sample volume from 10.0 to 50.0 mL; however, when the sample volume was above 50 mL, the extraction efficiency was reduced significantly. This may occur since a larger sample volume results in more adsorbent loss during the recovery process. Consequently, 50 mL of sample solution volume was selected.

#### 2.3.2. Optimization of MSPE- Fe_3_O_4_@SiO_2_@C18

##### Effect of pH

**Figure 8:**
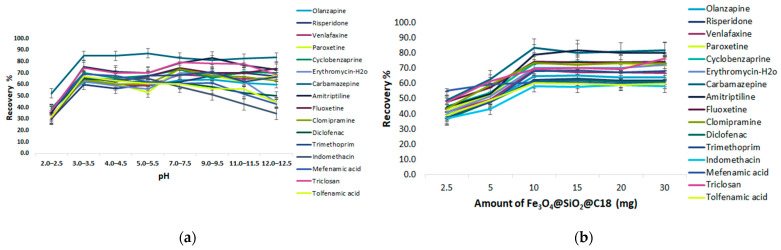

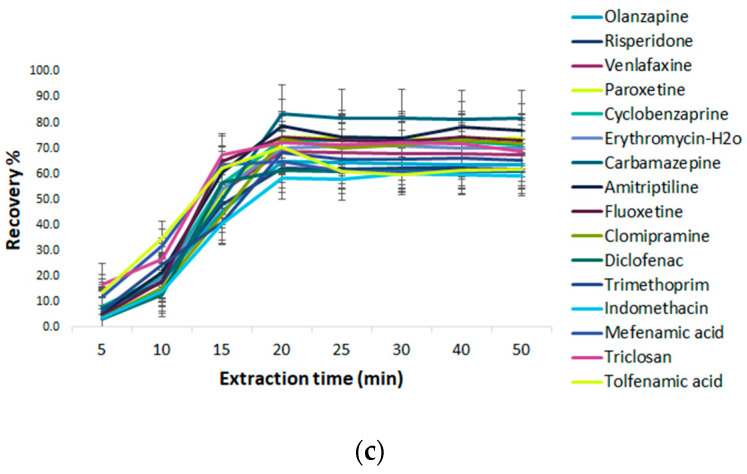
Effect of the extraction parameters of MSPE@C18 (**a**) pH, (**b**) amount of GO@Fe_3_O_4_, and (**c**) extraction time.

The investigated range of the pH, from 2.0 to 12.5, was studied by setting the extraction time at 60 min and using 1 mL of MeOH as a desorption solvent for 30 min. The target analytes selected for the sorption of C18@SiO_2_@Fe_3_O_4_ have a different pKa ranging from 3.88–15.52 and it was expected that the pH would have a different effect on them. Most analytes are basic compounds with amine groups in the structure, and pKa values from 8.0–9.0. The extraction efficiency started to increase above pH 2.0, while above pH 7.0 the compounds were almost constant, indicating the increasing partition of C18-MNPs due to the increasing van der Waals forces among C18 alkyl groups. The analytes are supposed to show a decreasing ionization tendency with an increase of pH, especially at an extremely alkaline pH. However, as seen in Figure 8a, the sorption of analytes is possible even at lower and neutral pHs, due to the possible residual silanol groups in the sorbent, which can exhibit hydrogen bonds or a dipolar interaction with the analytes in a weak acidic or neutral medium, and cationic exchange interactions in a strong acidic medium. The analytes with a pKa of 3.8–4.0 (diclofenac, indomethacin, mefenamic acid, triclosan, tolfenamic acid) showed similar sorption trends in sorbent. Specifically, they presented maximum adsorption at pH 3.0–3.5 as expected from the pKa values, since the analytes exist in molecular form in acidic pH, facilitating the extraction process. The ERs are constant when pH 7.0 is reached, with a slight decrease and minimum recovery of 58.1% (indomethacin) in this area. On the contrary, we observed important decreasing ERs in alkaline pH, due to the total ionization of the analytes and the less hydrophobic character of the compounds, which does not promote sorption in the C18 sorbent. In view of the normal pH circumstance of wastewater being close to neutral, pH 7.0 was chosen for the following experiments, since most analytes can maintain their original states and the recoveries of the extraction are satisfactory as well.

##### Amount of Fe_3_O_4_@SiO_2_@C18

The adsorbent amount was also investigated. Different amounts of the MNPs—C18 from 2.5 to 30 mg—were tested to optimize the amount of sorbent. As can be seen in the extraction efficiency profile shown in Figure 8b, the mean recovery for all the analytes increased with an increase in the amount of sorbent from 10 to 30 mg, while mefenamic acid kept a stable trend and thereafter leveled off. This could be explained by the fact that by increasing the amount of sorbent, the area required for the adsorption of the analytes is increased as well, up to a certain level. As can be deduced from Figure 8b, the application of 10 mg sorbent provided the most satisfactory recoveries for all the analytes. Therefore, 10 mg was the optimum amount and was used for the next experiments.

##### Effect of Extraction Time on Fe_3_O_4_@SiO_2_@C18 -MSPE

A short extraction time leads to the incomplete sorption of the target substance in the solution onto the adsorbent, nevertheless a long extraction time makes the MSPE process unnecessarily lengthy. Therefore, several experimental tests were performed in the range of 5–50 min. As can be seen in Figure 8c the extraction efficiency was significantly increased with a rise in stirring time from 5 to 20 min, after which, as the time prolonged, no remarkable increase in extraction efficiency was observed. Satisfactory extraction recoveries for the analytes were obtained at 20 min. These experimental data indicate that the sorption equilibriums were achieved quickly. It may be deduced that the large surface area of the C18 sorbent and the numerous C18 groups anchored in the magnetic nanoparticles resulted in a strong hydrophobic interaction between the analytes and the sorbent, making the distribution equilibrium easy to achieve in a brief time. Therefore, 20 min was chosen as the optimal extraction time and employed for the subsequent tests.

##### Desorption Conditions

**Figure 9:**
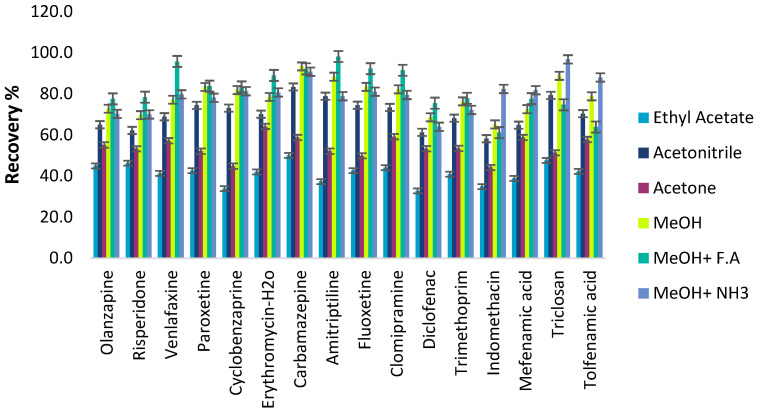
Effect of the desorption solvent on the recoveries of ECs.

A suitable desorbing solvent should effectively elute the adsorbed analytes with the minimum volume without damaging the nature of the adsorbent surface and with fewer interfering impurities coeluted. Several eluents, such as acetonitrile, methanol, acetone, and ethyl acetate, were evaluated to select the best eluent solvent for eluting the pharmaceutical compounds from the C18-MNPs. Their recovery efficiencies were evaluated under the same extraction and elution conditions and are depicted in Figure 9. Methanol exhibited the highest recoveries and the acidity or alkalinity in desorption solvent was further evaluated with the addition of formic acid and ammonium hydroxide solution in various concentrations (0–5% *v*/*v*). The results indicate that methanol acidified with 1.0% *v*/*v* formic acid (f.a) provided the highest elution capability for all the compounds in comparison with other solvents. Most analytes are in an ionic state in acidic FA solution, so reduced interaction occurs with non-polar C18 groups. Therefore, methanol containing 1% *v*/*v* f.a was chosen as the elution solvent.

##### Effect of Sample Volume

**Figure 10:**
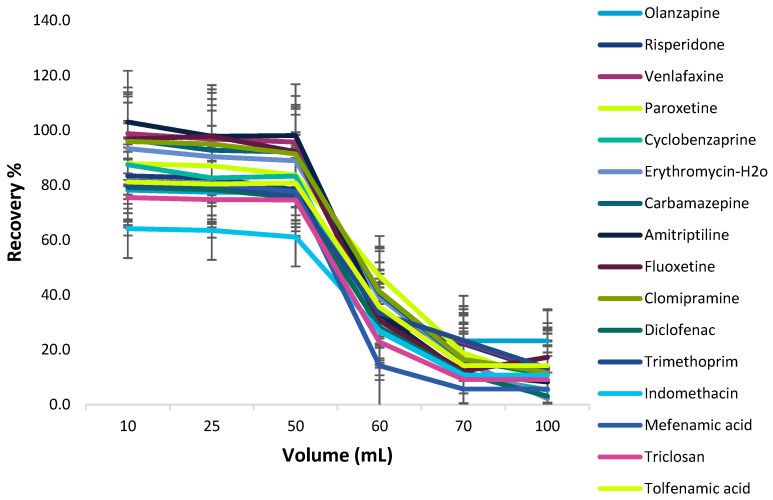
Effect of sample volume on Fe_3_O_4_@SiO_2_@C18-MSPE.

The possibility of enriching low concentrations of the target analytes from large volumes of the samples was examined by studying the effect of sample volume on the recovery of the analytes. The sample solution volume effect was assessed by treating 15 mg magnetic C18 nanoparticles with different sample volumes (10–100 mL) of the standard solutions, each of them containing 1 μg of analytes. As shown in Figure 10, quantitative recoveries of the target analytes were obtained with up to 50 mL of the sample solution, while above 50 mL the recoveries decreased. Hence, a sample volume of 50 mL was selected as the ideal volume for the extraction of the target analytes from C18 MNPs.

### 2.4. Reuse of Magnetic Sorbents

**Figure 11:**
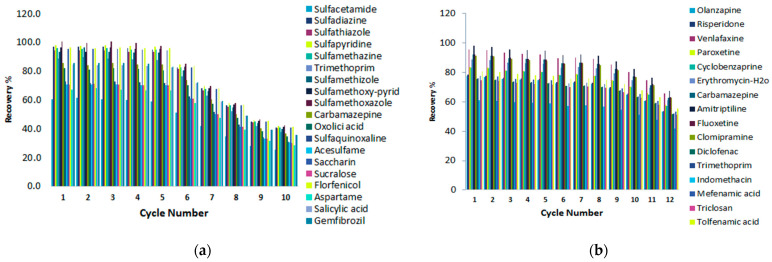
Reuse of the magnetic sorbents (**a**) Fe_3_O_4_@GO and (**b**) Fe_3_O_4_@SiO_2_@C18.

Reusability is one of the key factors for evaluating the stability and efficiency of the adsorbent. The sorption–desorption cycles were repeated 10 times when the graphene-based components were employed in the MSPE protocol, and 12 times when the C18 silica magnetic nanoparticles were applied. After each experiment cycle, the magnetic sorbents were isolated from the sample solution using an external magnet and washed three times with methanol. Afterwards they were dried under nitrogen before their reuse in the next cycle. As shown in Figure 11, the magnetic graphene composites could be effectively reused at least five times without a significant loss of the extraction efficiency. On the other hand, the regenerated C18 magnetic nanoparticles could be applied for more cycles. It can be seen that the percentage removal started to decrease significantly after 10 consecutive sorption cycles.

The optimized extraction parameters (Table 1) for both MSPE protocols were applied in a pooled sample of wastewater from two different sampling stations (WWTP of Ioannina and WWTP of the University Hospital of Ioannina) as well as in tap water, to evaluate the analytical performance of the two methods.

### 2.5. Validation of Magnetic Solid-Phase Extraction

The two methods (MSPE@GO and MSPE@C18) developed for the determination of the selected emerging contaminants in aqueous media were finally validated. Validation studies as well as the identification and confirmation of the target compounds were based on the quality control procedures established by the European Union (EU) regulations (EU Commission Decision, 2002) [29]. The validation procedures were conducted in two different samples, tap water and effluent wastewater, providing different performance criteria, such as sensitivity, linearity, precision and reproducibility, and accuracy. Matrix effect studies were also evaluated for both aqueous matrices.

#### 2.5.1. Accuracy

The accuracy of the developed method was checked by recovery experiments. Recoveries in the MSPE@GO method ranged from 58.4% to 102.6% for effluent water, while for tap water recoveries varied from 53.8 to 99.8% for all concentration levels. Recoveries below 60% were considered in the correction of the concentration in the quantification procedure. The evaluation of the accuracy experiments in the MSPE@C18 method concluded with satisfactory recoveries > 60% in all cases for the two matrices. In effluent water recoveries ranged from 61.1% for indomethacin to 98.5% for venlafaxine, while tap water provided equivalent results concerning the minimum and the maximum recovery value (64.9% for indomethacin and 99.5% for venlafaxine) (Table 2).

#### 2.5.2. Sensitivity and Linearity

The method detection limits (MDLs) and method quantification limits (MQLs) were determined, for tap water and effluent samples, as the minimum detectable and quantifiable amount of analyte with a signal-to-noise 3 and 10, respectively. The levels of the MDLs and MQLs varied depending on the aquatic matrix, and higher values were achieved for WWTP effluent water. Regarding MSPE@GO extraction, the MDLs in tap water ranged between 0.4 and 29.4 ng/L, while in effluent wastewater they ranged between 0.6 and 31.2 ng/L. The respective MQLS for the analyzed compounds presented variations from 1.2 to 90.5 ng/L for tap water and 1.8 to 98.7 ng/L for effluent wastewater with a higher value for sucralose.

In MSPE@C18, the MDLs and MQLs for tap water ranged from 0.3 to 6.6 ng/L and 0.9 to 19.8 ng/Lrespectively, with a lower detection limit for oxolinic acid. Concerning the effluent water, the applied method provided MDLs and MQLs in the range of 0.38–10.9 ng/Land 1.1–33.6 ng/L respectively with the lowest detection limit for oxolinic acid as well.

Linearity was investigated by establishing the adequate lineal range for each compound from MQLs of approximately 100 times MQL (100xMQL) for tap water and effluent wastewater. All the target compounds exhibited good linearity and the calibration curves showed, in all cases, coefficients of determination (R2) greater than 0.99.

#### 2.5.3. Precision

The precision expressed as repeatability (intra-day precision) and reproducibility (inter-day precision) in terms of relative standard deviation (RSDr and RSDR, respectively) was evaluated in tap water samples and effluent wastewater spiked at three concentration levels of MQL, 10xMQL and 100xMQL. Regarding the MSPE@GO method, the RSDr (*n* = 5) values for the intra-day analyses were in the range of 2.1–16.7% and the RSDR for the inter-day (*n* = 15) values were between 2.6% and 18.1% for tap water. As regards effluent water, the RSDs of the spiked samples were lower than 14% for all the target analytes with the exception of salicylic acid. The higher RSD of salicylic acid observed at the lowest concentration level can be attributed to the elevated concentrations found in the blank samples. The MSPE@C18 method showed good precision for tap water, since the RSDr values (*n* = 5) observed for the intra-day analyses were in the range of 3.8–17.6% in all cases, while the RSDR values (*n* = 15) over different days were below 18.2%. Slightly higher RSDs were displayed for effluent water with intra-day precision (*n* = 5) ranging from 3.9 to 23.1 and inter-day precision (*n* = 15) below 24.8% for all cases.

#### 2.5.4. Matrix Effect (ME)

**Figure 12:**
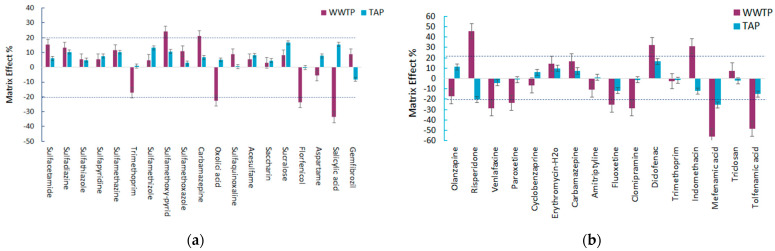
Matrix effect for the selected pharmaceuticals in the different aqueous matrices after (**a**) MSPE@Fe_3_O_4_@GO and (**b**) MSPE@SiO_2_@C18.

The ME was evaluated for the different aquatic matrices and the results are shown in Figure 12a,b. The ME was calculated according to Equation (1) [30,31,32].

(1) $$Matrix\;effect\;\left(\%\right)=\left(\frac{slope\;matrix-matched}{slope\;standard\;solution}-1\right)\times 100$$

From the calculated matrix effect results of the samples extracted with MSPE@GO, it can be assumed that in all the studied matrices there is evidence of the matrix effect. In general, the matrix effect in tap water was not significant or negligible and was expressed in most cases as signal enhancement (−20 < ME < 20). On the other hand, in effluent water from the total 19 target compounds studied, 14 of them presented a positive matrix effect expressed as signal enhancement and 5 of them showed signal suppression. However, for the majority of the analytes a low matrix effect was observed (−20 < ME < 20) with the only exceptions being five pharmaceutical compounds: oxolinic acid, florfenicol, salicylic acid, carbamazepine, and sulfamethoxy-pyridazine, which presented a medium matrix effect. The positive matrix values of carbamazepine (21.1%) and sulfamethoxy-pyridazine (24.3%) indicated the enhancement of the signal, while the negative values of oxolinic acid, florfenicol, and salicylic acid implied signal suppression. Salicylic acid showed the strongest matrix effect in this studied data set, with a maximum signal suppression of −33.7%.

The results of the matrix effect for MSPE@C18 showed variations concerning the aqueous matrix as well as the signal enhancement/signal suppression. In tap water, most of the tested compounds exhibited a low matrix effect, with the only exceptions being risperidone and mefenamic acid, which showed a medium matrix effect expressed as ion suppression with ME values of −20.3% and −25.3%, respectively. These high percentages of ME for drinking water are still low compared with the corresponding percentages for effluent water. The values of the matrix effect for effluent water were higher than 20% or less than −20% for 9 out of the 16 compounds under study. Five of them showed ion suppression (venlafaxine, paroxetine, clomipramine, and tolfenamic acid), while three of the target analytes showed ion enhancement (risperidone, indomethacin, and diclofenac). Specifically, risperidone, diclofenac, venlafaxine, paroxetine, fluoxetine, clomipramine, and tolfenamic acid were subjected to a medium matrix effect, while mefenamic acid was suppressed with a negative value of −55.8%, indicating a high matrix effect.

Evaluating the general ME observed for the different aquatic matrices in both the methods developed, it was noticed that the ME was less pronounced (<20%) in drinking water for all the selected pharmaceuticals, while for WWTP effluent, more than 50% of the pharmaceuticals had a ME higher than 20%. In fact, due to the high complexity of wastewater, the matrix effects were more pronounced in this category. In addition, it can also be assumed that the matrix effect is a compound dependent phenomenon, since there are compounds that belong in the same class of pharmaceuticals but still present diverse matrix effects.

### 2.6. Analysis of the Real Samples

The applicability of the developed methods, MSPE@GO and MSPE@C18, was evaluated on effluent wastewater samples from the hospital facilities of Ioannina, as well as from the municipal WWTP located in the same city. Three pooled effluent waters of three consecutive sampling days were analyzed (triplicate analysis). Matrix matched-calibration curves were used for the quantification of the target analytes. Table 3 summarizes the average calculated concentration (after three consecutive samplings).

Briefly, the analysis revealed the occurrence of 13 and 19 multi-class emerging contaminants in urban and hospital effluent, respectively. Eleven target compounds were quantified in urban wastewater, while 13 were above the quantification limit in hospital effluent. Thirteen analytes of interest were not detected at any sample station. The mean concentrations of the detected compounds ranged from 12.4 to 10,324.6 ng/L and 12.9 to 487.5 ng/L for effluent urban water and hospital effluent, respectively. The highest mean concentration was observed for the artificial sweetener acesulfame, 10,324.6 ngL^−1^, with higher orders of magnitude compared to the other studied compounds, confirming its intense presence, which has also been mentioned in other relative studies [33,34]. Acesulfame has also been noted in many monitoring studies as a chemical marker for domestic wastewater input [35,36,37,38]. On the other hand, from the hospital effluent analysis, the maximum mean concentration presented was for sulfamethoxazole, one of the most broad-spectrum antibiotics consumed worldwide. Its occurrence in wastewater effluent from hospital facilities makes sense and is highlighted by other studies too [39,40,41,42,43,44]. High concentration levels in both sampling stations were also observed for salicylic acid, an ubiquitous compound in wastewater studies in Greece, since it is the main metabolite of aspirin (acetylsalicylic), a popular first-line OTC (over the counter) anti-inflammatory drug. The related work of Kosma et al., 2014 [45] in hospital units confirms these findings in urban and hospital wastewater.

Finally because of the complexity of wastewaters as a matrix, any false-positives/negatives must be investigated in MS experiments [46]. The identification was based on the criteria proposed by the EU Commission Decision 2002/657/EC in combination with FDA guidelines that take full advantage of the capabilities of modern HRMS instruments [47,48], in addition to the last specific update for the identification of small molecules [49]. Specifically, in order to comply with the established criteria, the identification was based on the comparison of the retention times (<0.1 min), the accurate mass of the m/z (Δ < 5 ppm), and the existence of at least one fragment ion with a high mass accuracy (Δ < 5 ppm).

## 3. Materials and Methods

### 3.1. Materials and Chemicals

All the pharmaceutical active compounds and sweeteners were of high purity (>95%) and the stock solutions were prepared in LC–MS grade solvents of methanol (MeOH), acetonitrile (AcN), and formic acid (f.a), purchased from Fisher Scientific (Leicester, UK). Ultrapure water (resistivity of 18.2 MΩ-cm) was obtained by using an Evoqua purification system (Evoqua, Pittsburg, PA, USA).

For the synthesis of the magnetic nanoparticles, ferric chloride (FeCl_3_) was purchased from Fluka (Milwaukee, WI, USA) and ferrous chloride tetrahydrate (FeCl_2_·4H_2_O) from Fischer Scientific (Berlin, Germany). Tetraethyl orthosilicate (TEOS) and trimethoxy(octadecyl)silane (99%) were purchased from Sigma-Aldrich. Sulfuric acid (96%), phosphoric acid (85%), potassium permanganate (99%), hydrogen peroxide (30% *w*/*w*), hydrochloric acid (37%), ethanol (99%), and sodium nitrate (99%) were provided by Sigma-Aldrich (Athens, Greece). Hydrazinium hydroxide (~100% for synthesis) was purchased from Merck (Merck, Darmstadt, Germany). Natural graphite (99.99% purity, 20 µm, from Merck) was used as precursor of graphene oxide (GO).

### 3.2. Equipment

The morphological characterization of graphene, graphene oxide nanosheets, and their magnetic analogs was conducted with scanning electron microscope (SEM) (JSM-7001F, Japan). The corresponding SEM images of the C18 magnetic nanoparticles were obtained using a JOEL microscope (JSM-5600, JEOL, Tokyo, Japan) after gold coating. The crystal structure characterization was conducted by X-ray diffraction (XRD) on a Bruker D8-advance X-ray diffractometer at 40 kV and 40 mA using Cu Kα (λ = 1.5406 Å) radiation and the sample was scanned from 5° to 60°, in steps of 0.02° (2*θ*), at a rate of 2 s per step. The magnetic properties of the respective nanomaterials were examined on a vibrating magnetometer (LakeShore 7300, Westerville, OH, USA) at room temperature.

The chromatographic analysis was conducted on an Accela UHPLC system (Thermo Fisher Scientific, Bremen, Germany) consisting of an Accela autosampler (model 2.1.1) and an Accela quaternary gradient UHPLC pump (model 1.05.0900). The chromatographic system was coupled to a hybrid LTQ Orbitrap XL Fourier transform mass spectrometer (Thermo Fisher Scientific, Bremen, Germany). The linear ion trap (LTQ) part of the hybrid MS system was equipped with an Ion Max electrospray ionization probe, operating in the positive and negative ionization mode.

### 3.3. UHPLC–LTQ Orbitrap MS Analysis

The strategy followed for the evaluation of the operational conditions of the UHPLC Orbitrap-MS was based on a previously published article of the authors [50]. The chromatographic conditions were evaluated for positive (PI) and negative (NI) ionization. The LC separation was achieved using a reversed-phase Hypersil Gold C18 analytical column (100 mm, 2.1 mm, 1.9 µm), purchased from Thermo Scientific, and maintained at 35 °C. The mobile phase employed for the separation consisted of water (A) and methanol (B), both containing 0.1% *v*/*v* formic acid. Two different elution gradients were applied for positive and negative ionization. For PI, the gradient started at 95% mobile phase A and was maintained for 1 min; the next minute mobile phase B was increased to 70% followed by an increase to 100% for 3 min, where it remained stable for an additional 2 min. Afterwards, the mobile phase was restored to the initial conditions of 95% A and maintained over 3 min for the re-equilibration of the column. The total running time was 10 min with a flow rate of 250 μL min^−1^ and the injection volume set at 10 μL. A gradient program with slight modifications was used for the separation of compounds ionized in negative mode: 90% of mobile phase (A) was used from 0–0.5 min, followed by consecutive linear declines to 30% (A) from 0.5 to 2.0 min, to 10% (A) from 2.0 to 3.0, and 5% (A) from 3.0 to 3.9. In the 4.5 min duration of the total run the percentage of methanol (B) increased to 100% and this composition was maintained for half a minute. Finally, the column was re-equilibrated with 90% (A) from 5.1 to 8.0 min. The mobile phase was delivered at a flow rate of 200 μL min^−1^ in a 35 °C thermostatted column. A 20 μL aliquot of the sample was injected.

Along with the chromatographic conditions, the operational parameters of the Orbitrap MS (ESI parameters, mass resolving power, AGC target, tube lens, injection time) were evaluated for the confirmation of analytes at trace concentration levels. Instrumental detection limits, mass accuracy, and fragment ions and their corresponding collision energy for all emerging contaminants of the study in positive and negative ionization modes are included in Tables S2 and S3 in the supplementary material. The qualification and quantification analyses were performed in full scan accurate mass spectra at a high resolution of 60,000 as profile data, while at the same time, a ddMS2 experiment was running for identification and confirmation purposes. MS2 spectra were acquired only for the precursor ions defined from an inclusion list with target analytes, at a resolution of 15,000. The ESI-MS settings were analyte-specific, and their optimization was evaluated based on a previous study of the authors [50]. Briefly, for the PI mode the following source parameters were applied: capillary temperature 320 °C; capillary voltage; spray voltage, 4.0 kV; tube lens voltage, 90 V; sheath gas, 35 au; auxiliary gas, 10 au. In full-scan MS mode the following parameters were used: mass range, 120–1000; automatic gain control target (AGC), 5 × 10^5^, and the maximum injection time (IT) was set to 100 ms, and the number of micro scans to be performed was set at 1 scan s^−1^.

In negative ionization mode (NI) the applied parameters were as follows: capillary temperature, 320 °C; capillary voltage, 30 V; spray voltage, 2.7 kV; tube lens voltage, −90 V; sheath gas, 10 au; auxiliary gas, 7 au. In full-scan acquisition mode the scan range was set at 120–600 *m*/*z*, the automatic gain control target (AGC) was set at a target value of 4 × 10^04^, and the maximum injection time (IT) was 80 ms. The number of microscans to be performed was set at 1 scan s^−1^.

Furthermore, the MS/MS scans were applied by targeting the automatic gain control (AGC) at 2 × 10^5^ and 2 × 10^4^ ions for PI and NI, respectively, while the maximum injection time (IT) was set at 50 ms for both polarity modes. The mass tolerance window was set to 5 ppm. Several values of normalized collision energies (NCEs) were assessed for the selection of the optimum fragmentation pattern for each target compound. The optimized parameters for the full MS/dd-MS2 analysis are listed in Tables S2 and S3. Data processing was employed with Xcalibur 2.1 (Thermo Electron, San Jose, CA, USA).

### 3.4. Preparation of Magnetic Materials

#### 3.4.1. Preparation of Fe_3_O_4_@SiO_2_@C18

The preparation of the silica based magnetic nanomaterials involved three steps: (a) the synthesis of Fe_3_O_4_, which was used as the magnetic core of the MNPs; (b) the surface coating with silica following the Stober process; and (c) the final modification with the C18 as-bonded phase consisted of the octadecyl alkyl chain. Details of the synthetic procedure for each step of the entire process are reported in another publication of this research group [17].

#### 3.4.2. Preparation of Fe_3_O_4_@GO

Following a typical procedure, graphene oxide (GO) was produced from pure graphite powder according to the modified method reported by Hummers and Offerman [51,52]. Briefly, 1.0 g of graphite and 0.5 g of NaNO_3_ were added into 23 mL of 0 °C concentrated H_2_SO_4_. Next, 3 g of KMnO4 was added gradually with continuous stirring and cooling, while at the same time the temperature of the mixture was maintained below 20 °C. Then, the ice-bath was removed, and the obtained mixture was stirred at 25 °C for 30 min. After that time, 46 mL of distilled water was added slowly to reach a temperature of 98 °C and the mixture was maintained at that temperature for 15 min. The resultant reaction was terminated with the addition of 140 mL of distilled water followed by 10 mL of 30% H_2_O_2_ aqueous solution. The GO was collected by centrifugation. The solid materials were washed consecutively with water and ethanol. The resultant materials were dried under a vacuum overnight at 25 °C to obtain GO [53].

##### Synthesis of Magnetic Graphene Oxide (mGO)

The synthesis of magnetic graphene oxide was based on a previous study by Chatzimitakos et al. [26]. Magnetic graphene oxide was prepared by the co-precipitation of Fe^2+^ and Fe^3+^ in the presence of GO in an alkaline solution according to the following procedure [26]. To obtain a dispersion solution, 0.5 g of GO was exfoliated to 100 mL of ultrapure water with ultrasonication for 1 h. The dispersion was heated at 80 °C and degassed under nitrogen flow for 10 min. Then, 20 mL of ultrapure water containing 0.7 g of ferric chloride (FeCl3) and 0.42 g of ferrous chloride (FeCl24 H_2_O) was added slowly to the above dispersion and the mixture was stirred vigorously under continuous nitrogen flow. Next, 6 mL of a concentrated ammonium hydroxide solution of 25% *v*/*v* was added instantly and the mixture was further stirred for 30 min. The mixture was left to cool at room temperature. Then, the magnetic GO was isolated using a neodymium magnet and washed three times with ultrapure and three times with ethanol. The nanomaterial was dried in an oven at 70 °C overnight, ground to a fine powder with the aid of a mortar, and stored at room temperature [26,53].

##### Synthesis of Magnetic Reduced Graphene Oxide (mrGO)

In order to obtain Fe_3_O_4_@rGO (mrGO) a typical procedure with the direct addition of a reducing agent was followed [26]. Using ultrasonication, 50 mg of synthesized mGO t was dispersed in 20 mL ultrapure water. Afterwards, 0.25 mL of hydrazinium hydroxide (N_2_H_5_OH) was added to the dispersion (final concentration, 0.1 mol L^−1^) with constant stirring to result in a black solution. Vigorous stirring of the dispersion was continued for 24 h at 70 °C, under reflux. Finally, the precipitate was isolated using a neodymium magnet (Nd–Fe–B), washed several times with deionized water, followed by three times with ethanol, and dried under a vacuum at 60 °C for 12 h [54].

### 3.5. Application of MSPE for the Extraction of ECs from Hospital and Urban Wastewater

**Figure 13:**
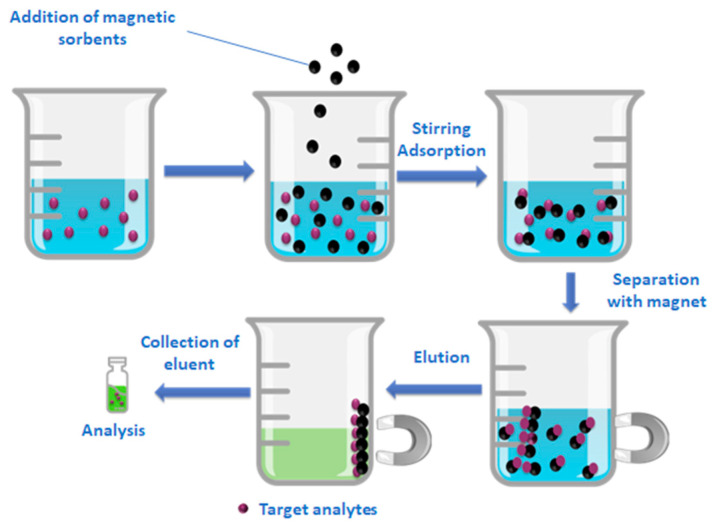
Schematic procedure of MSPE.

Three effluent water samples were collected from the municipal sewage plant of Ioannina city located in the Epirus region in north-west Greece (WWTP-u), as well as from Ioannina University hospital (WWTP-h) over three consecutive days. The water samples were stored at 4 °C in the dark and analyzed within 48 h of collection. The samples were pooled and pretreated according to the following MSPE protocols with the employment of the aforementioned synthesized materials before UHPLC-Orbitrap MS analysis. Three replicates were performed for each pooled sample. A schematic diagram of the MSPE procedure is illustrated in Figure 13.

#### 3.5.1. MSPE@C18

The MSPE procedure with the use of C18 MNPs was conducted as follows. First, 10 mg of C18 nanocomposites was introduced into a beaker and activated with 5 mL of MEOH. Then, 50 mL of a standard solution or water sample was added. The mixture was stirred for 20 min to reach the sorption equilibrium, and once the extraction was complete, the target analytes adsorbed onto the magnetic nanocomposites were separated from the liquid phase with a neodymium magnet placed at the bottom of the beaker. Next, the supernatant was poured out and finally, the adsorbed analytes were eluted and desorbed from the sorbent by a 1 min sonication while applying 1 mL methanol containing 1% (*v*/*v*) formic acid over two cycles (2 × 1 mL). Then, placing again the magnet, the eluate was obtained and transferred to a vial to be dried with a stream of nitrogen and afterwards was reconstituted in 250 μL of the initial mobile phase.

#### 3.5.2. MSPE@GO

Firstly, 15 mg of the Fe_3_O_4_@GO was rinsed and activated in 5 mL of methanol and then dispersed in 50 mL of a fortified/nonfortified aqueous sample whose pH had been adjusted to 3.0–3.5 with 1 M HCl. The mixture was stirred for 15 min to accomplish the extraction of the target analytes. Subsequently, an Nd–Fe–B strong magnet was deposited at the bottom of the beaker to hold the magnetic graphene composites which had already extracted the analytes. After about 5 min, the solution became clear and the supernatant was discarded, while the target analytes were desorbed in two replicates from the Fe_3_O_4_@GO MNPs with 2.0 mL methanol containing 1% (*v*/*v*) ammonia by sonication for 1.0 min. Afterwards the magnet was placed again on the outside bottom of the beaker and the desorption solution was collected using a micropipette. The collected desorption solution was evaporated to dryness under a gentle N_2_ flow at 30 °C and was reconstituted with 250 μL of the initial mobile phase.

## 4. Conclusions

Magnetic materials based on silic-C18 and graphene oxide were successfully synthesized, characterized, and introduced as selective and effective sorbents for the determination of a multiclass group of emerging contaminants in wastewater. After the optimization of the extraction parameters, the proposed techniques along with UHPLC-Orbitrap-MS analysis yielded high recoveries, low detection limits, linearity of R^2^ > 0.99, and satisfactory repeatability for the selected analytes. The applicability of the method in urban and hospital effluent provided reliable measurements of ECs in wastewaters in terms of accuracy and selectivity. The overall performance of the magnetic solid-phase extraction in combination with LC-Orbitrap MS/MS in wastewater analysis suggests a promising tool for wastewater monitoring campaigns and studies about environmental impact.

## Figures and Tables

**Table 1:** Optimal parameters of MSPE with Fe_3_O_4_@GO and Fe_3_O_4_@SiO_2_@C18.

Optimized Extraction Conditions	MSPE–Fe_3_O_4_@GO	MSPE –Fe_3_O_4_@SiO_2_@C18
pH	3.0	7.0
Sorbent Amount Extraction Time Elution Solvent Elution volume–Desorption Time	15 mg	10 mg
	15 min	20 min
	MeOH + 1%NH3	MeOH + 1% f.a
	2 × 2 mL–2 × 60 s	2 × 1 mL–2 × 30 s

**Table 2:** Parameters indicating the performance of the analytical methods MSPE-GO, MSPE-C18. Method detection and quantification limits (MDL, MQL), linearity(R^2^), and matrix effect in all matrices studied.

	MSPE@Fe_3_O_4_@GO									MSPE@Fe_3_O_4_@SiO_2_@C18							
	TAP WATER				EFFLUENT					TAP WATER				EFFLUENT			
Compound	R^2^	MDL ng/L	MQL ng/L	ME %	R^2^	MDL ng/L	MQL ng/L	ME %		R^2^	MDL ng/L	MQL ng/L	ME %	R^2^	MDL ng/L	MQL ng/L	ME %
Sulfacetamide	0.9991	3.8	12.9	5.9	0.9973	9.8	32.8	15.4	Olanzapine	0.9991	1.5	4.4	11.1	0.9948	1.80	5.8	−17.0
Sulfadiazine	0.9987	3.2	10.4	10.3	0.9967	5.1	12.9	13.1	Risperidone	0.9951	0.6	1.8	−20.3	0.9912	1.45	4.9	45.9
Sulfathiazole	0.9990	3.9	13.9	4.8	0.9970	4.3	14.6	5.3	Venlafaxine	0.9990	0.3	0.9	−4.2	0.9988	0.65	1.8	−28.7
Sulfapyridine	0.9994	4.1	11.3	7.6	0.9975	3.9	13.5	5.3	Paroxetine	0.9968	1.9	5.8	−0.9	0.9933	3.84	11.2	−23.3
Sulfamethazine	0.9997	1.1	3.4	10.1	0.9949	1.3	3.5	11.6	Cyclobenzaprine	0.9992	0.5	1.5	5.9	0.9987	0.49	1.6	−6.7
Trimethoprim	0.9998	0.4	1.2	0.8	0.9978	1.1	2.1	−17.2	Ery-H_2_O	0.9998	2.3	7.0	9.7	0.9991	2.35	7.2	14.4
Sulfamethizole	0.9989	1.9	6.4	13.1	0.9959	2.0	6.8	4.9	Carbamazepine	1.0000	0.3	0.9	7.3	0.9994	0.38	1.1	16.6
Sulfamethoxy-pyrid	0.9995	0.8	2.5	10.5	0.9928	1.2	3.3	24.3	Amitriptyline	0.9958	1.3	3.8	1.1	0.9948	1.39	4.1	−10.6
Sulfamethoxazole	0.9998	0.9	2.7	2.9	0.9978	0.9	2.9	10.8	Fluoxetine	0.9981	1.3	3.8	−11.8	0.9969	1.68	5.1	−25.1
Carbamazepine	1.0000	0.9	2.6	6.7	0.9993	1.0	3.1	21.1	Clomipramine	0.9992	1.4	4.1	−1.2	0.998	2.01	6.1	−28.9
Oxolici acid	0.9993	1.2	3.0	5.0	0.9981	0.6	1.8	−22.6	Diclofenac	0.9993	6.6	19.8	16.4	0.9928	10.9	33.6	32.0
Sulfaquinoxaline	0.9987	0.7	2.0	0.6	0.9931	0.9	2.9	8.8	Trimethoprim	0.9998	0.7	2.1	−1.5	0.999	0.71	1.9	−2.7
Acesulfame	0.9992	10.8	32.4	8.1	0.9941	10.4	34.8	5.4	Indomethacin	0.9987	5.1	15.4	−12.2	0.9954	7.0	21.8	30.9
Saccharin	0.9993	11.2	30.7	4.5	0.9964	10.0	35.1	3.1	Mefenamic acid	0.9994	5.6	16.9	−25.3	0.9977	5.9	19.8	−55.8
Sucralose	0.9990	29.4	90.5	16.5	0.9973	31.2	98.7	8.0	Triclosan	0.9987	2.3	6.8	−2.3	0.9952	2.5	7.1	7.6
Florfenicol	0.9996	0.7	1.9	−0.1	0.9944	0.6	2.0	−23.5	Tolfenamic acid	0.9999	3.7	11.0	−14.8	0.9991	4.9	15.4	−48.6
Aspartame	0.9981	22.4	69.9	7.7	0.9964	30.2	93.9	−5.6									
Salicylic acid	0.9933	10.3	29.4	15.3	0.9913	11.8	37.8	−33.7									
Gemfibrozil	0.9900	5.9	18.8	−8.1	0.9922	9.4	29.4	8.9									

**Table 3:** Mean concentrations (*n* = 3) of target ECs in urban (WWTP-u) and hospital (WWTP-h) effluent.

Concentration (ngL^−1^)		
Analyte	WWTP-u	WWTP-h
Acesulfame	10,324.6	157.6
Amitriptyline	15.1	25.8
Aspartame	<MDL	<MDL
Carbamazepine	133.2	478.0
Clomipramine	<MDL	<MQL
Cyclobenzaprine	<MDL	<MDL
Diclofenac	152.9	156.9
Erythromycin-H_2_O	<MQL	14.9
Florfenicol	<MDL	<MDL
Fluoxetine	32.8	23.1
Gemfibrozil	<MDL	<MDL
Indomethacin	<MDL	<MDL
Mefenamic acid	<MDL	<MQL
Olanzapine	<MDL	<MDL
Oxolinic acid	<MDL	<MDL
Paroxetine	<MQL	40.4
Risperidone	<MDL	<MDL
Saccharin	<MQL	<MQL
Salicylic acid	723.6	451.8
Sucralose	1246.1	<MQL
Sulfacetamide	<MDL	189.5
Sulfadiazine	<MDL	<MQL
Sulfamethazine	<MDL	<MDL
Sulfamethizole	<MDL	<MDL
Sulfamethoxazole	189.6	487.5
Sulfamethoxy-pyridazine	<MDL	<MDL
Sulfapyridine	<MDL	109.3
Sulfaquinoxaline	12.4	<MDL
Sulfathiazole	<MDL	<MDL
Tolfenamic acid	<MDL	<MDL
Triclosan	115.4	110.9
Trimethoprim	<MDL	12.9
Venlafaxine	145.2	165.2

<MDL = below detection limit, <MQL = below quantification limit.

## Data Availability

Not applicable.

## Supplementary Materials

The following supporting information can be downloaded at: https://www.mdpi.com/article/10.3390/molecules28052277/s1, Table S1: Selection of magnetic sorbents for the extraction of investigated analytes; Figure S1: Extracted Ion Chromatogram, XIC of standard solution of 5 μg/L in UHPLC–LTQ/Orbitrap of target analytes (a) positive ionization, (b) negative ionization; Table S2: Parameters for full MS/dd-MS2 Orbitrap analysis (Positive Ionization); Table S3: Parameters for full MS/dd-MS2 Orbitrap analysis (Negative Ionization); Table S4: LogP parameter of target analytes.
