# Supplementary material for: Magnetic Solid-Phase Extraction Based on Silica and Graphene Materials for Sensitive Analysis of Emerging Contaminants in Wastewater with the Aid of UHPLC-Orbitrap-MS
Source: Molecules. 2023 Feb 28;28(5):2277. doi: 10.3390/molecules28052277 (PMC10005528; doi:10.3390/molecules28052277)
Supplement: Supplementary file 1 [file molecules-28-02277-s001.zip › molecules-2193347-supplementary.pdf]

molecules

Supplementary Materials

Magnetic Solid-Phase Extraction Based on Silica and Graphene Materials for Sensitive Analysis of Emerging Contaminants in Wastewater with the aid of UHPLC-Orbitrap-MS

Table S1. Selection of magnetic sorbents for the extraction of investigated analytes.

Compounds	MSPE_Fe_3O_4@GO	MSPE_Fe_3O_4@SiO_2@C18
Acesulfame	✓	
Amitriptyline		✓
Aspartame	✓	
Carbamazepine	✓	✓
Clomipramine		✓
Cyclobenzaprine		✓
Diclofenac		✓
Erythromycin-H2O		✓
Florfenicol	✓	
Fluoxetine		✓
Gemfibrozil	✓	
Indomethacin		✓
Mefenamic acid		✓
Olanzapine		✓
Oxolinic acid	✓	
Paroxetine		✓
Risperidone		✓
Saccharin	✓	
Salicylic acid	✓	
Sucralose	✓	
Sulfacetamide	✓	
Sulfadiazine	✓	
Sulfamethazine	✓	
Sulfamethizole	✓	
Sulfamethoxazole	✓	
Sulfamethoxy-pyridazine	✓	
Sulfapyridine	✓	
Sulfaquinoxaline	✓	
Sulfathiazole	✓	
Tolfenamic acid		✓
Triclosan		✓
Trimethoprim	✓	✓
Venlafaxine		✓

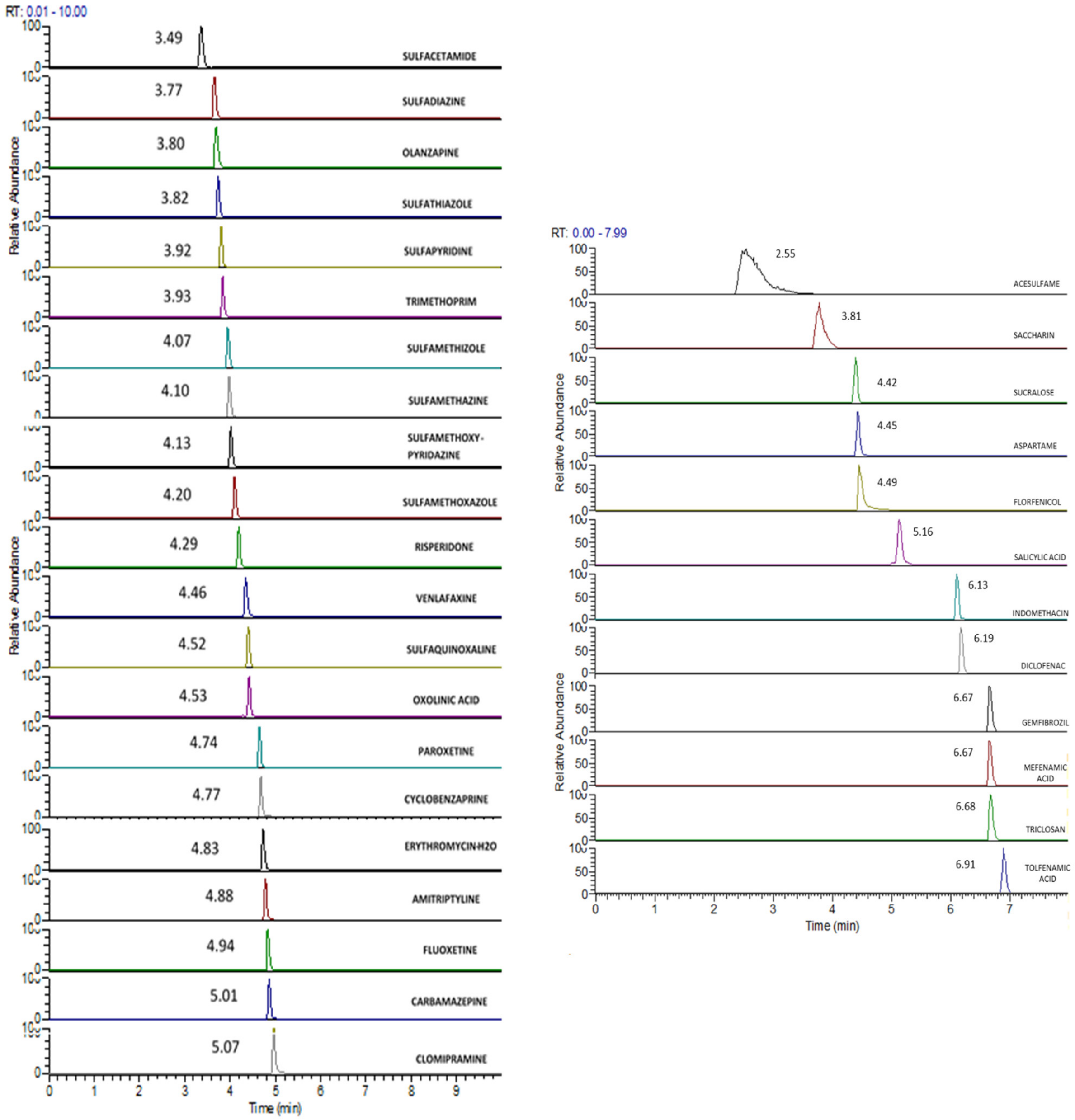

Figure S1. Extracted Ion Chromatogram, XIC of standard solution of 5 μg/L in UHPLC–LTQ/Orbitrap of target analytes a) positive ionization, b) negative ionization.

Table S2. Parameters for full MS/dd-MS² Orbitrap analysis (Positive Ionization).

COMPOUND	Rt(min)	Elemental formula	Theoretical mass (m/z)	Empirical mass (m/z)	RDB	Δ (ppm)	IQL (μgL-1)	FRAGM. ION	ELE-MENTAL FORMULA	NCE
Sulfacetamide	3.49	$C_8H_{11}N_2O_3S$+	215.0485	215.0486	4.5	0.514	0.44	108.0448<br>92.0500<br>156.0112	C_6HNO+<br>C_6H_6N+<br>$C_6H_6NO_2S$+	30
Sulfadiazine	3.77	$C_{10}H_{11}N_4O_2S$+	251.0597	251.0598	7.5	0.307	0.22	156.0112<br>92.0489	$C_6H_6NO_2S$+<br>$C_6H_6NO_2S$+	30
Olanzapine	3.80	$C_{17}H_{21}N_4S$+	313.1481	313.1481	9.5	-0.140	0.23	256.0902<br>230.0745	$C_{14}H_{14}N_3S$+<br>$C_{12}H_{12}N_3S$+	35
Sulfathiazole	3.82	$C_9H_{10}N_3O_2S_{2+}$	256.0209	256.0211	6.5	0.021	0.24	156.0112<br>108.0440	C_6HNO_2S+<br>C_6H_6NO+	30
Sulfapyridine	3.92	$C_{11}H_{12}N_3O_2S$+	250.0645	250.0645	7.5	0.105	0.07	156.0112<br>108.0440	C_6HNO_2S+<br>C_6H_6NO+	30

Trimethoprim	3.93	$C_{14}H_{19}N_4O_{3+}$	291.1452	291.1453	7.5	0.457	0.18	184.0867<br>230.1161<br>261.0980	$C_{11}H_{10}N_3$+<br>$C_{12}H_{14}N_4O$+<br>$C_{12}H_{13}N_4O_3$+	30
Sulfamethizole	4.07	$C_9H_{11}N_4O_2S_{2+}$	271.0318	271.0319	6.5	0.393	0.17	156.0113<br>108.0447<br>65.0393	$C_6H_6NO_2$+<br>C_6H_6NO+<br>C_5H_5+	35
Sulfamethazine	4.10	$C_{12}H_{15}N_4O_2S$+	279.0910	279.0909	7.5	-0.441	0.16	92.050<br>123.0662	C_6H_6N+<br>$C_6H_9N_3$+	30
Sulfamethoxy-pyridazine	4.13	$C_{11}H_{13}N_4O_3S$+	281.0703	281.0703	7.5	0.045	0.23	156.0113<br>108.0430<br>92.0486	$C_6H_6NO_2S$+<br>C_6H_6NO+<br>C_6H_6N+	20

Table S2. Parameters for full MS/dd-MS² Orbitrap analysis, *(continued)*.

COMPOUND	Rt(min)	Elemental formula	Theoretical mass (m/z)	Empirical mass (m/z)	RDB	Δ (ppm)	IQL (μgL-1)	FRAGM. ION	ELE-MENTAL FORMULA	NCE
Sulfamethoxazole	4.20	$C_{10}H_{12}N_3O_3S$+	254.0594	254.0592	6.5	-0.742	0.22	188.0818<br>156.0113<br>147.0789	$C_{10}H_{10}N_3O$+<br>$C_6H_6NO_2S$+<br>$C_8H_9N_3$+	20
Risperidone	4.29	$C_{23}H_{28}FN_4O_{2+}$	411.2191	411.2190	11.5	-0.197	0.44	191.1171	$C_{11}H_{15}N_2O$+	35
Venlafaxine	4.46	$C_{17}H_{28}NO_{2+}$	278.2115	278.2117	4.5	0.878	0.42	121.0643<br>260.2006	C_8H_9O+<br>$C_{17}H_{26}NO$+	25
Sulfaquinoxaline	4.52	$C_{14}H_{13}N_4O_2S$+	301.0754	301.0754	10.5	0.090	0.44	156.0113<br>108.0440	C_6HNO_2S+<br>C_6H_6NO+	30
Oxolinic Acid	4.53	$C_{13}H_{12}NO_{5+}$	262.0710	262.0709	8.5	-0.378	0.07	244.1905	$C_{13}H_{10}NO_4$+	25
Paroxetine	4.74	$C_{19}H_{21}O_3NF$+	330.1500	330.1502	9.5	0.611	0.06	192.1180<br>151.0387	$C_{12}H_{15}NF$+<br>$C_8H_7O_3$+	35
Cyclobenzaprine	4.77	$C_{20}H_{22}N$+	276.1747	276.1749	10.5	0.810	0.16	58.0659<br>84.0814	C_3H_8N+<br>$C_5H_{10}N$+	30
Erythromycin-H_2O	4.83	$C_{37}H_{66}NO_{12}$	716.4580	716.4591	5.5	1.601	0.14	158.1175<br>558.3635	$C_8H_{16}O_2N$+<br>$C_{26}H_{54}O_{12}$	40
Amitriptiline	4.88	$C_{20}H_{24}N$+	278.1903	278.1905	9.5	0.624	0.09	233.1322<br>191.0854<br>155.0854<br>117.0695	C_8H_{17}+<br>$C_{15}H_{11}$+<br>$C_{12}H_{11}$+<br>C_9H_9+	30
Fluoxetine	4.94	$C_{17}H_{19}F_3NO$+	310.1413	310.1415	7.5	0.563	0.06	148.1119<br>247.0918	$C_{10}H_{14}N$+<br>$C_4H_{18}FN_3$+	15
Carbamazepine	5.01	$C_{15}H_{13}N_2O$	237.1022	237.1024	10.5	0.677	0.16	194.0964<br>220.0756<br>192.0808	$C_{14}H_{12}N$+<br>$C_{15}H_{10}NO$+<br>$C_{14}H_{10}N$+	35
Clomipramine	5.07	$C_{19}H_{24}ClN_{2+}$	315.1623	315.1627	8.5	1.418	0.10	86.0940<br>58.059	$C_5H_{12}N$+<br>C_3H_8N+	30

Table S3. Parameters for full MS/dd-MS2 Orbitrap analysis (Negative Ionization).

COMPOUND	Rt(min)	Elemental for-mula	Theoretical mass (m/z)	Empirical mass (m/z)	RDB	Δ (ppm)	IDL (μgL-¹)	IQL (μgL-¹)	FRAGM. ION	ELEMENTAL FORMULA	NCE
Acesulfame	2.55	$C_4H_4NO_4S^-$	161.9867	161.9868	3.5	0.916	0.94	3.13	77.9642<br>82.0285	NO_2S-<br>C_4H_4NO-	30
Saccharin	3.79	$C_7H_4NO_3S^-$	181.9917	181.9919	6.5	0.896	0.07	0.24	105.9592<br>61.9693	CNO_3S-<br>NOS-	30
Sucralose	4.41	$C_{12}H_{18}Cl_3O_8^-$	395.0073	395.0072	2.5	-0.187	0.19	0.63	359.0299	$C_{12}H_{17}Cl_2O_8$	30
Aspartame	4.44	$C_{14}H_{17}N_2O_5^-$	293.1143	293.1141	7.5	-0.665	0.07	0.24	261.0880	$C_{13}H_{13}$N2O4-	15
Florfenicol	4.47	$C_{12}H_{13}Cl_2FNO_4S^-$	355.9932	355.9936	5.5	1.164	0.02	0.06	335.9869<br>218.9890	$C_{12}H_{12}Cl_2NO_4S$-<br>$C_8H_8ClO_3S$-	35
Salicylic acid	5.14	$C_7H_5O_3^-$	137.0244	137.0250	5.5	4.252	0.05	0.16	93.00334<br>65.0384	C_6H_5O-<br>C_5H_5-	30
Indomethacin	6.12	$C_{19}H_{15}ClNO_4^-$	356.0659	356.0675	12.5	-2.281	0.30	0.91	312.0796	$C_{18}H_{15}NO_2Cl$-	
Diclofenac	6.19	$C_{14}H_{10}Cl_2NO_2^-$	294.0094	294.0089	9.5	-1.725	0.22	0.74	250.0193	$C_{13}H_{10}Cl_2N$-	35
Gemfibrozil	6.67	$C_{15}H_{21}O_3^-$	249.1496	249.1489	5.5	-2.881	0.38	1.25	121.0659	C_8H_9O-	25
Mefenamic Acid	6.67	$C_{15}H_{14}NO_2^-$	240.103	240.1026	9.5	-1.674	0.07	0.23	196.1133<br>240.1029	$C_{14}H_{14}N$-<br>$C_{15}H_{14}NO_2$-	20
Triclosan	6.68	$C_{12}H_6Cl_3O_2^-$	286.9439	286.9433	8.5	-2.042	0.18	0.59	161.2632	$C_6H_3Cl_2O$-	25
Tolfenamic Acid	6.91	$C_{14}H_{11}ClNO_2^-$	260.0484	260.0480	9.5	-1.460	0.23	0.77	216.0580	$C_{14}H_{11}ClNO_2$-	20

* Rt: Retention time; IDL: Instrumental Detection Limit.

Table S4. LogP parameter of target analytes.

Compounds	logP
Acesulfame	-0.552
Amitriptyline	4.810
Aspartame	-2.218
Carbamazepine	2.766
Clomipramine	4.883
Cyclobenzaprine	4.613
Diclofenac	4.259
Erythromycin	2.596
Florfenicol	0.670
Fluoxetine	4.173
Gemfibrozil	4.387
Indomethacin	3.530
Mefenamic acid	5.398
Olanzapine	3.388
Oxolinic acid	1.353
Paroxetine	3.148
Risperidone	2.628
Saccharin	0.449
Salicylic acid	1.977
Sucralose	-0.465
Sulfacetamide	-0.300
Sulfadiazine	0.387
Sulfamethazine	0.650
Sulfamethizole	0.214
Sulfamethoxazole	0.791
Sulfamethoxy-pyridazine	0.466
Sulfapyridine	1.009
Sulfaquinoxaline	1.552
Sulfathiazole	0.975
Tolfenamic acid	5.488
Triclosan	4.982
Trimethoprim	1.284
Venlafaxine	2.739

Data concerning obtained from international database *https://chemaxon.com/*.
